# Selectivity of mycoinsecticides and a pyrethroid to the egg parasitoid *Cleruchoides noackae* (Hymenoptera: Mymaridae)

**DOI:** 10.1038/s41598-020-71151-2

**Published:** 2020-09-03

**Authors:** Maurício Magalhães Domingues, Luciane Katarine Becchi, Simone Graziele Moio Velozo, Amanda Rodrigues de Souza, Leonardo Rodrigues Barbosa, Marcus Alvarenga Soares, José Eduardo Serrão, José Cola Zanuncio, Carlos Frederico Wilcken

**Affiliations:** 1 Faculdade de Ciências Agronômicas, Universidade Estadual Paulista (UNESP), Campus de Botucatu, Botucatu, São Paulo 18610-034 Brasil; 2 Empresa GERDAU, Três Marias, Minas Gerais 39205-000 Brasil; 3 Empresa Brasileira de Pesquisa Agropecuária, Embrapa Florestas, Colombo, Paraná 83411-000 Brasil; 4 Programa de Pós-Graduação em Produção Vegetal, Universidade Federal dos Vales do Jequitinhonha e Mucuri (UFVJM), Diamantina, Minas Gerais 39100-000 Brasil; 5 Departamento de Biologia Geral, Universidade Federal de Viçosa, Viçosa, Minas Gerais 36570-900 Brasil; 6 Departamento de Entomologia/BIOAGRO, Universidade Federal de Viçosa, Viçosa, Minas Gerais 36570-900 Brasil

**Keywords:** Animal behaviour, Entomology

## Abstract

Plants of the genus *Eucalyptus*, cultivated in many countries, have great importance for the world economy. In Brazil, this culture occupies a total of 5.7 million hectares, but native and exotic insect pests can reduce its productivity. *Thaumastocoris peregrinus* Carpintero & Dellapé (Hemiptera: Thaumastocoridae), an exotic Australian pest, damages *Eucalyptus* plants. Biological control using the egg parasitoid *Cleruchoides noackae* Lin & Huber (Hymenoptera: Mymaridae), Heteroptera predators and entomopathogenic fungi, such as *Beauveria bassiana* and *Metarhizium anisopliae*, have potential for managing *T. peregrinus*. Chemical insecticides, including bifenthrin and acetamiprid + bifenthrin, also control this insect. The compatibility of chemical and biological control methods favors integrated pest management. The objective of this study was to evaluate the selectivity of commercial products based on *B. bassiana*, *M. anisopliae* and the chemical bifenthrin on the parasitoid *C. noackae* and its parasitism on *T. peregrinus* eggs. The selectivity test followed the standards recommended by the International Organization for Biological Control (IOBC). *Beauveria bassiana* has selectivity to parasitism as well as viability, but was slightly harmful to *C. noackae* adults; *M. anisopliae* was innocuous to adults and to the viability of the offspring of this parasitoid, but it reduced the parasitism rate; and bifenthrin did not show selectivity to this parasitoid.

## Introduction

The area of commercially planted forests in the world increased from 167.5 to 277.9 million hectares from 1990 to 2015^1^. Brazil presently has 5.7 million hectares of *Eucalyptus* plantations with 24%, 17% and 15% of them in the states of Minas Gerais, São Paulo and Mato Grosso do Sul, respectively. The wood from these plantations is mainly destined for the pulp industry, with a production of 21 million tons in 2018^2,3^.

Insect pests of Australian origin detected in planted forests during the last three decades on a global scale may reduce *Eucalyptus* productivity^4^. The bronze bug, *Thaumastocoris peregrinus* Carpintero & Dellapé (Hemiptera: Thaumastocoridae), was first detected in Brazil in 2008 in the states of São Paulo and Rio Grande do Sul, and has since dispersed to other *Eucalyptus*-producing states^5^. This insect develops and produces fertile offspring on most *Eucalyptus* plantations in Brazil^6^. *Thaumastocoris peregrinus* perforates and causes silvering, tanning, drying and defoliation from *Eucalyptus* plants^7^.

Biological control is the most widely-used method for managing *T. peregrinus*^8^. This method includes the egg parasitoid *Cleruchoides noackae* Lin & Huber (Hymenoptera: Mymaridae), imported from Australia^8,9^, the predators *Atopozelus opsimus* Elkins (Hemiptera: Reduviidae)^10^ and *Supputius cincticeps* Stäl (Heteroptera: Pentatomidae)^11,12^ and entomopathogenic fungi^13,14^. *Beauveria bassiana* and *Metarhizium anisopliae*, registered commercially^15^ and considered to offer reduced risks, are the most studied entomopathogenic fungi^16,17^. The chemical insecticides bifenthrin and acetamiprid + bifenthrin are also used to control *T. peregrinus* in *Eucalyptus* plantations^15^.

Natural enemies are important in pest control in planted forests, justifying the search for compatible microbial and chemical products^18^. The mycoinsecticides and chemical insecticides must have selectivity to the pest natural enemies^19,20^ in order to maintain the effectiveness of the combined use of these methods.

The objective of this study was to evaluate the effect of commercial products based on *B. bassiana* and *M. anisopliae* and of the chemical insecticide bifenthrin on the egg parasitoid *C. noackae* and on its parasitism on *T. peregrinus* eggs.

## Results

### Mortality of *Cleruchoides noackae* adults

**Figure 1:**
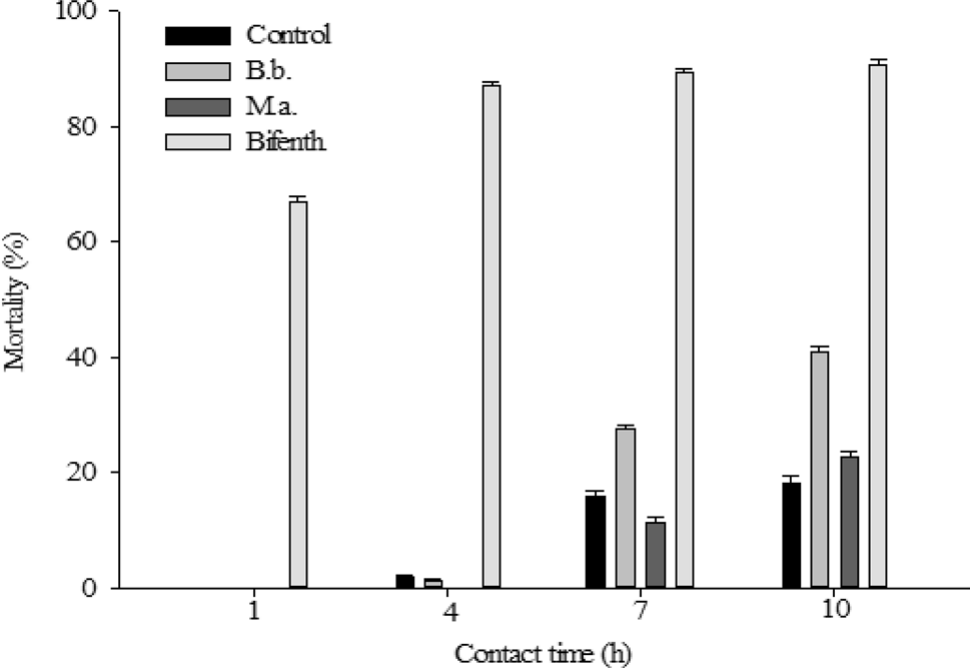
Mortality (%) of *Cleruchoides noackae* (Hymenoptera: Mymaridae) adults, over the time of contact in hours (h) with the biological insecticides *Beauveria bassiana* (B.b.) and *Metarhizium anisopliae* (M.a.) and the chemical bifenthrin (Bifenth.) (temp.: 25 ± 1ºC, RH: 70 ± 10% and photophase: 12 h).

The mortality of *C. noackae* adults was higher with bifenthrin after the first and tenth hours of exposure to this chemical, with 67% and 90.6%, respectively. Biological products based on *B. bassiana* and *M. anisopliae* caused mortality of 40.8% and 22.6%, respectively, of *C. noackae* adults after 10 h of exposure, higher than the control, distilled and autoclaved water, which was 18% (Fig. 1). Bifenthrin was moderately harmful, *B. bassiana* was mildly harmful, and *M. anisopliae* was innocuous to *C. noackae* adults, presenting a reduction in the beneficial ability of the parasitoid [%E = 100 − (average for each insecticide/average for the percentage in the control treatment) × 100] of 90.60, 40.80 and 22.60, respectively, according to IOBC classification (Table 1).

**Table 1:** Mortality (%) of *Cleruchoides noackae* (Hymenoptera: Mymaridae) adults, through contact with the biological insecticides *Beauveria bassiana* (B.b.) and *Metarhizium anisopliae* (M.a.) and the chemical bifenthrin (Bifenth.) as treatments (Treat.) (temp.: 25 ± 1ºC, RH: 70 ± 10% and photophase: 12 h) and classes of these products (Cl.).

Treat	Contact time (h)						
	1	4	7	10		%E^a^	Cl^b^
Control	0.0 ± 0.00Aa	2.0 ± 0.25Aa	15.9 ± 0.86Aa	18.0 ± 1.52Aa		0.00	-
B.b	0.0 ± 0.00Aa	1.3 ± 0.20Aa	27.6 ± 0.40Ba	40.8 ± 1.16Bb		40.80	2
M.a	0.0 ± 0.00Aa	0.0 ± 0.00Aa	11.3 ± 1.11Aa	22.6 ± 1.08Aa		22.60	1
Bifenth	67.0 ± 0.93Ab	87.0 ± 0.66Ab	89.4 ± 0.58Ab	90.6 ± 0.80Ac		90.60	3

Means followed by the same uppercase letter per line or lower case per column do not differ according to the Tukey test (p ≤ 0.05).

^a^%E: reduction in the beneficial capacity of the parasitoid.

^b^Cl—class 1—harmless (E < 30%), class 2—slightly deleterious (30% ≤ E ≤ 79%), class 3—moderately harmful (80% ≤ E ≤ 99%), class 4—harmful (E > 99%).

### Parasitism and viability of *C. noackae* on treated *T. peregrinus* eggs

Parasitism by *C. noackae* differed between treatments (ANOVA; F = 4.9259, P = 0.01862), with a lower value for the bifenthrin than in the control, *B. bassiana* or *M. anisopliae*. Bifenthrin was moderately harmful (%E = 88.89), *B. bassiana* innocuous (%E = 2.8), and *M. anisopliae* slightly deleterious (%E = 36.12) to *C. noackae* parasitism on treated *T. peregrinus* eggs. The *C. noackae* viability ranged from 96 to 100% between treatments, with all the products being classified as harmless (%E = 0 to 5) (Table 2).

**Table 2:** Parasitism (Paras.) and viability (Viab.) (mean ± SD) (%) and reduction in the beneficial capacity of the parasitoid *Cleruchoides noackae* (Hymenoptera: Mymaridae) (% E) on *Thaumastocoris peregrinus* (Hemiptera: Thaumastocoridae) eggs treated with different insecticides (temp.: 25 ± 1 ºC, RH: 70 ± 10% and photophase: 12 h) and class of these products (Cl.).

Treatments	Paras. (%)	%E	Cl	Viab. (%)	%E	Cl
Control	72 ± 15.94a	0.00	1	100 ± 0.00a	0.00	-
*Beauveria bassiana*	70 ± 8.94a	2.80	1	96 ± 4.47a	5.00	1
*Metarhizium anisopliae*	46 ± 15.68ab	36.12	2	100 ± 0.00a	0.00	1
Bifenthrin	8 ± 4.90b	88.89	3	100 ± 0.00a	0.00	1

Means followed by the same lowercase letter per column do not differ according to the Tukey test (p ≤ 0.05).

### Viability of *C. noackae* in eggs parasitized and treated after one and 10 days of exposure to the insecticides

*C. noackae* viability in *T. peregrinus* eggs treated with the insecticides after one day of parasitism differed between treatments (ANOVA; F = 4.301, P = 0.0126), with a lower value for the bifenthrin than in the control and with the product *B. bassiana* having a value similar to that of *M. anisopliae* (Table 3). Bifenthrin reduced the viability of this natural enemy on parasitized eggs after 10 days (ANOVA; F = 6.460, P = 0.0018). This chemical was slightly harmful (%E = 30.11 and 34.08), and *M. anisopliae* (%E = 13.02 and 1.68) and *B. bassiana* (%E = 4.75 and 4.60) innocuous, after one and 10 days, respectively, for the parasitoid *C. noackae* (Table 3).

**Table 3:** Viability (%) and reduction of the beneficial capacity (% E) of the parasitoid *Cleruchoides noackae* (Hymenoptera: Mymaridae) on *Thaumastocoris peregrinus* (Hemiptera: Thaumastocoridae) eggs treated with the fungi *Beauveria bassiana* and *Metarhizium anisopliae* and with the chemical bifenthrin after one and 10 days of parasitism (temp.: 25 ± 1° C, RH: 70 ± 10% and photophase: 12 h) and class of these insecticides (Cl.).

Treatments	Viability (%)					
	1 Day	%E^a^	Cl	10 days	%E^a^	Cl
Control	99.36 ± 4.4Aa	0.00	1	94.92 ± 9.6Aa	0.00	-
*Beauveria bassiana*	94.64 ± 7.3Aa	4.75	1	90.55 ± 12.9Aa	4.60	1
*Metarhizium anisopliae*	86.42 ± 13.4Aab	13.02	1	93.33 ± 14.9Aa	1.68	1
Bifenthrin	69.44 ± 16.1Ab	30.11	2	62.57 ± 23.2Ab	34.08	2

Means followed by the same uppercase letter per line or lower case per column do not differ according to the Tukey test (p ≤ 0.05).

^a^%E: reduction in the beneficial capacity of the parasitoid.

## Discussion

The entomopathogenic fungi tested were chosen according to the knowledge and use of these microorganisms in biological control, as well as their reduced environmental impact^21^. Selectivity tests show the low impact of products on non-target organisms, and allow the recommendation of combinations of mycoinsecticides and chemical insecticides to manage harmful organisms.

The higher mortality of *C. noackae* adults produced by bifenthrin shows that this chemical is moderately harmful, like most pyrethroids that keep the sodium channels of the neuron membranes open and reach the insect peripheral and central nervous systems^22^. At the cellular level, these compounds stimulate the neurons to produce repetitive discharges, leading to membrane depolarization and synaptic disorders^23^. Cyanide pyrethroids cause hypersensitivity, choreoathetosis, tremors, paralysis and insect mortality^24,25^. The classification of *B. bassiana* as slightly harmful to *C. noackae* adults may be related to the production of secondary metabolites, such as organic acids involved in the infection process and linear and cyclic peptidic toxins such as beauvericin from the mycelium of this fungus^26,27^. The lack of *Metarhizium anisopliae* toxicity to *C. noackae* adults is similar to that reported with this fungus on *Cotesia flavipes* Cameron (Hymenoptera: Braconidae)^28^ and *Trichogramma galloi* Zucchi (Hymenoptera: Trichogrammatidae)^29^. *Metarhizium anisopliae* is important for biological control because some isolates may be highly specific and others infect a wide host range^30^. The mortality of *C. noackae* adults at shorter intervals is due to the reduced longevity of this parasitoid: 0.8 to 1.6 days when they were not fed^31^ and 3.5 days when they were fed with undiluted honey^32^, evidencing the importance of the evaluations during the first day of this natural enemy life.

The findings of lower *C. noackae* parasitism in *T. peregrinus* eggs treated with bifenthrin agree with reports that this compound is slightly to moderately harmful to *Trichogramma chilonis* Ishii, *Trichogramma ostriniae* Pang & Chen*,* and *Trichogramma dendrolimi* Matsumura (Hymenoptera: Trichogrammatidae)^33^ and harmful to *Encarsia formosa* Gahan, *Encarsia pergandiella* Howard (Hymenoptera: Aphelinidae)^34^, *Theocolax elegans* Westwood (Hymenoptera: Pteromalidae)^35^, *Eretmocerus mundus* Mercet (Hymenoptera: Aphelinidae)^36^ and *Telenomus podisi* Ashmead (Hymenoptera: Platygastridae)^37^, a common impact related to the pyrethroid action mode^23^. The effect of *M. anisopliae*, being slightly harmful to *C. noackae* parasitism on *T. peregrinus* eggs, differs from that reported for *Spalangia cameroni* Perkins (Hymenoptera: Pteromalidae), without reduction of its total female reproduction^38^, and innocuous to *Trichogramma pretiosum* Riley and *T. galloi* (Hymenoptera: Trichogrammatidae) parasitism^29,39^. These differences may be due to the specificity of cyclic peptidic toxins, called destruxins, related to *M. anisopliae* pathogenicity^40^. The lack of reduction of parasitism by *B. bassiana* is similar to that observed with *T. pretiosum*^39^, evidencing the selectivity of this fungus to natural enemies. The similar *C. noackae* viability on *T. peregrinus* eggs between treatments demonstrates that the products tested are innocuous to the development of this parasitoid in eggs of this host.

The lower *C. noackae* viability with bifenthrin, sprayed on *T. peregrinus* eggs at one and 10 days after parasitism, classified as slightly deleterious, differs from the classification of this chemical as having extremely low toxicity for the parasitoids *Eretmocerus tejanus* Rose & Zolnerowich and *E. mundus* (Hymenoptera: Aphelinidae), when applied at five and 14 days after parasitism^41^. This may be related to differences in the host development stage, since *C. noackae* is protected inside *T. peregrinus* eggs^42^, not allowing its direct contact with the insecticide. On the other hand, this differs from the effect on the larval parasitoids *E. tejanus* and *E. mundus*^41^ with a higher exposure to the chemical. The classification of mycoinsecticides as innocuous to *C. noackae* viability on *T. peregrinus* eggs parasitized at one and 10 days agrees with that observed for *Palmistichus elaeisis* Delvare & LaSalle, *Tetrastichus howardi* Olliff and *Trichospilus diatraeae* Cherian & Margabandhu (Hymenoptera: Eulophidae)^43^ and for *Telenomus remus* Nixon (Hymenoptera: Platygastridae)^44^, due to the specificity of the entomopathogenic fungi^45^ without impact on the development of egg parasitoids.

*Beauveria bassiana* and *M. anisopliae*, with high selectivity and low impact through contact with adults and in the parasitism and offspring of *C. noackae*, respectively, have potential for joint use with this parasitoid in pest management programs. However, these mycoinsecticides should be applied 3 days after releasing this parasitoid, avoiding contact with their adults at the time of parasitism. Bifenthrin, the first chemical insecticide registered to control *T. peregrinus*^15^, cannot be used with the *C. noackae* egg parasitoid to manage this pest.

*Beauveria bassiana*-based mycoinsecticides have selectivity to parasitism and viability and are slightly harmful to *C. noackae* adults; *Metarhizium anisopliae* was innocuous to adults and to the viability of this natural enemy offspring, but it reduced *C. noackae* parasitism on *T. peregrinus*; bifenthrin did not show selectivity in all bioassays.

## Methods

### Place of study

The work was carried out at the Laboratory of Biological Control of Forest Pests (LCBPF), Department of Plant Protection, School of Agricultural Sciences, Campus of Botucatu, São Paulo, Brazil, at 25 ± 1 ºC, 70 ± 10% relative humidity and photophase of 12 h.

### Rearing *Thaumastocoris peregrinus*

*Thaumastocoris peregrinus* adults were collected on two-year-old *Eucalyptus grandis* × *E. urophylla* plants at the FCA/UNESP and taken to the laboratory for mass rearing^46^.

Branches of the hybrid *Eucalyptus urophylla* var. *platyphylla* (clone 433) were collected from two-year-old trees, and arranged in bouquets with their bases in 250-ml Erlenmeyer flasks with water on a rectangular plastic tray (40 cm long × 35 cm wide × 8 cm high) to mass rear *T. peregrinus* in the laboratory. These bouquets were changed every three or four days depending on the need and leaf conditions. On the day of the exchange, the oldest and driest bouquets were placed next to new ones to facilitate the insect migration to the latter^46^.

### Rearing the parasitoid *Cleruchoides noackae*

*Thaumastocoris peregrinus* eggs, parasitized by *C. noackae*, were obtained from the LCBPF. Paper towel strips (1.5 cm wide × 15.0 cm long) were arranged in the upper portion of the leaves of the *T. peregrinus* breeding bouquets to obtain their eggs. *Cleruchoides noackae* were multiplied with *T. peregrinus* eggs with two to three days of age in transparent polystyrene bottles (7.5 cm high × 3.0 cm diameter).

Newly emerged *C. noackae* adults were transferred with a brush to another transparent polystyrene flask with paper towel strips with two- to three-day-old eggs obtained from the *T. peregrinus* rearing*. Cleruchoides noackae* were fed with 50% honey solution in filter paper strips (7.0 cm high × 1.5 cm wide)^47^.

### Selectivity test

The selectivity of mycoinsecticides and the bifenthrin-based insecticide to *C. noackae* adults and to their parasitism was evaluated in four treatments (Table 4), according to the protocol of the IOBC with the standard test cage^48^.

**Table 4:** Active principle (AP) and trademark (TM), manufacturer (Man.), concentration (Conc.), dose and formulation (For.) wettable powder (WP) and emulsifiable concentrate (EC) of the biological insecticides, based on *Beauveria bassiana* (*B. bassiana*) and *Metarhizium anisopliae* (*M. anisopliae*), and the chemical bifenthrin used in the selectivity tests with the parasitoid *Cleruchoides noackae* (Hymenoptera: Mymaridae).

AP	TM	Man	Conc	Dose	For
*B. bassiana*	BOVERIL® WP PL63	KBS	1.0 × 10^8^ conidia/g	2 kg/ha	WP
*M. anisopliae*	METARRIL ® WP E9	KBS	1.0 × 10^8^ conidia/g	0.5 kg/ha	WP
Bifenthrin	CAPTURE 400 EC	FMC	400 g/L	100 ml/ha	EC
Control	–	–	–	–	–

*KBS* Koppert Biological Systems, *FMC* FMC Química do Brasil Ltda.

One ml per replication of the biological and chemical products was applied in a Potter Tower on the surface of the cages designed according to the standard described by the IOBC and on parasitized or non-parasitized *T. peregrinus* eggs. Three bioassays were performed.

The first test evaluated the indirect action in the mortality of the parasitoid, exposed by contact to the biological and chemical products, using 100 new individuals per treatment, in five replications of 20 individuals each (per cage). The control had only distilled and autoclaved water. The parasitoids were released in the treated cages and their mortality, after contact with the treated surface, was evaluated.

The second bioassay evaluated the direct action on parasitism and the viability of *C. noackae* on *T. peregrinus* eggs treated with the insecticides, with five replications per treatment and 10 eggs, each one offered to a pair of the parasitoids per cage. Paper towel strips with one-day-old *T. peregrinus* eggs were treated with the insecticides, dried at room temperature and offered to each *C. noackae* couple for 24 h.

The third bioassay evaluated the *C. noackae* viability with the products. One-day-old *T. peregrinus* eggs, exposed to each *C. noackae* couple for 24 h, were treated in a Potter Tower after one and 10 days post-parasitism, respectively. Five replications with 10 eggs each were used per treatment (Table 4) and age after parasitism (one and 10 days), totaling 400 eggs.

### Data evaluation

Mortality, parasitism and viability (%) of *C. noackae* were evaluated. Mortality of this parasitoid was evaluated after the first hour of contact with the insecticides and then every three hours until completing 10 h, due to its reduced longevity. Parasitism and viability of *C. noackae* were evaluated after 13 days of parasitism (parasitoid cycle), considering emerged and retained parasitoids and non-parasitized and infertile eggs. The percentage of reduction in parasitoid beneficial ability was calculated for each of the analyzed variables (survival, parasitism and viability; %E) with the equation: %E = [100 − (average for each insecticide/average for the percentage in the control treatment) × 100] to classify the products according to IOBC standards: class 1—innocuous (E < 30%); class 2—slightly deleterious (30 ≤ E ≤ 79%); class 3—moderately harmful (80 ≤ E ≤ 99%); and class 4—harmful (E > 99%)^48^.

The design was completely randomized, the data submitted to variance analysis and the means compared by the Tukey test at 5% probability using the R Studio software.
